# Flexor Pollicis Longus Tendon Rupture After Volar Plating of a Distal Radius Fracture: Pronator Quadratus Plate Coverage May Not Adequately Protect Tendons

**Published:** 2011-11-09

**Authors:** Emile N. Brown, Scott D. Lifchez

**Affiliations:** Department of Plastic Surgery, Johns Hopkins Bayview Medical Center, Baltimore, MD

## Abstract

**Objective:** The senior author previously reported a case of rupture of the flexor policis longus tendon after volar plating of a distal radius fracture. We hypothesized that restoration of the pronator quadratus to its native position after plating might prevent this problem. **Methods:** The authors report a new case of irritation of the flexor policis longus 2.5 years after volar plating of a distal radius fracture. The plate was in good contact with the bone, and the pronator quadratus had been restored to its native position. Despite this, there was a partial-thickness laceration of the tendon over the plate and a defect in the pronator quadratus muscle which had been between the plate and the tendon. **Results:** The patient was treated with removal of the plate and repair of the tendon. She never lost function of the flexor policis longus tendon and has full function of her hand. **Conclusions:** The authors believe that restoration of the pronator quadratus to its native position after volar plating of a distal radius fracture does protect the overlying flexor tendons. Even with this step, tendon irritation can still occur. The authors advise their patients at the time of discharge from initial treatment of their distal radius fracture to be vigilant for any evidence of flexor tendon irritation and to return for evaluation if they have any suspicion of this.

Over the past decade, volar plating of distal radius fractures has become increasingly popular. Compared to dorsal plate fixation where the extensor tendons are in direct contact with the plate, the volar approach has a theoretical advantage in reducing complications of tendon irritation given the greater distance between the flexor tendons and the volar surface of the radius.^1^ However, there remains a risk of flexor tendon irritation with volar plate fixation, despite recent advances in plate design.^2^

The senior author previously reported a case of flexor policis longus (FPL) rupture in a 65-year-old woman associated with volar plating of a distal radius fracture, necessitating removal of the plate and tendon repair.^3^ In that patient, a second-generation, low-profile, fixed-angle, volar locking plate had been placed by an outside surgeon. During exploration, the plate was found to be fully in contact in bone with no prominent edges or screw heads.

Orbay^1^ has advocated restoration of the pronator quadratus to its native position after volar plating of the radius, providing a layer of vascularized tissue between the plate and the flexor tendons. In our previously reported patient, this had not been done. We hypothesized that tendon irritation might have been avoided by reapproximation of the pronator quadratus over the plate.

## CLINICAL CASE

We now present the case of a 75-year-old right hand dominant woman who fell down the stairs in her home and impacted her left hand in extension against a wall. She was found to have a left intra-articular distal radius fracture with greater than 25 degrees of dorsal tilt. She initially underwent closed reduction and splinting; however, subsequent imaging 1 week later revealed loss of reduction. Therefore, we took her to the operating room 2 weeks after the initial injury and performed open reduction and internal fixation with a second-generation, low-profile, fixed-angle, volar locking plate (Acumed, Hillsboro, Oregon). The pronator quadratus muscle was restored to its native position and provided coverage of the entire plate. The patient did well postoperatively and achieved union and full recovery of wrist and hand function.

**Figure 1:**
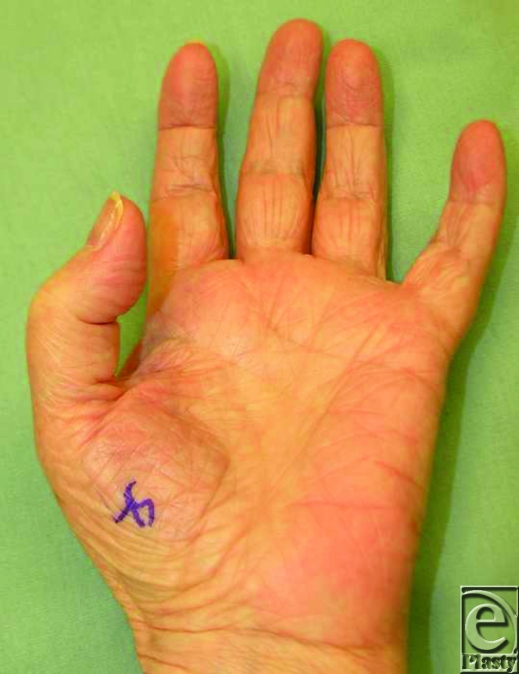
The patient retained ability to flex the left thumb interphalangeal at the time of presentation but complained of a grinding sensation at the wrist when she did.

**Figure 2:**
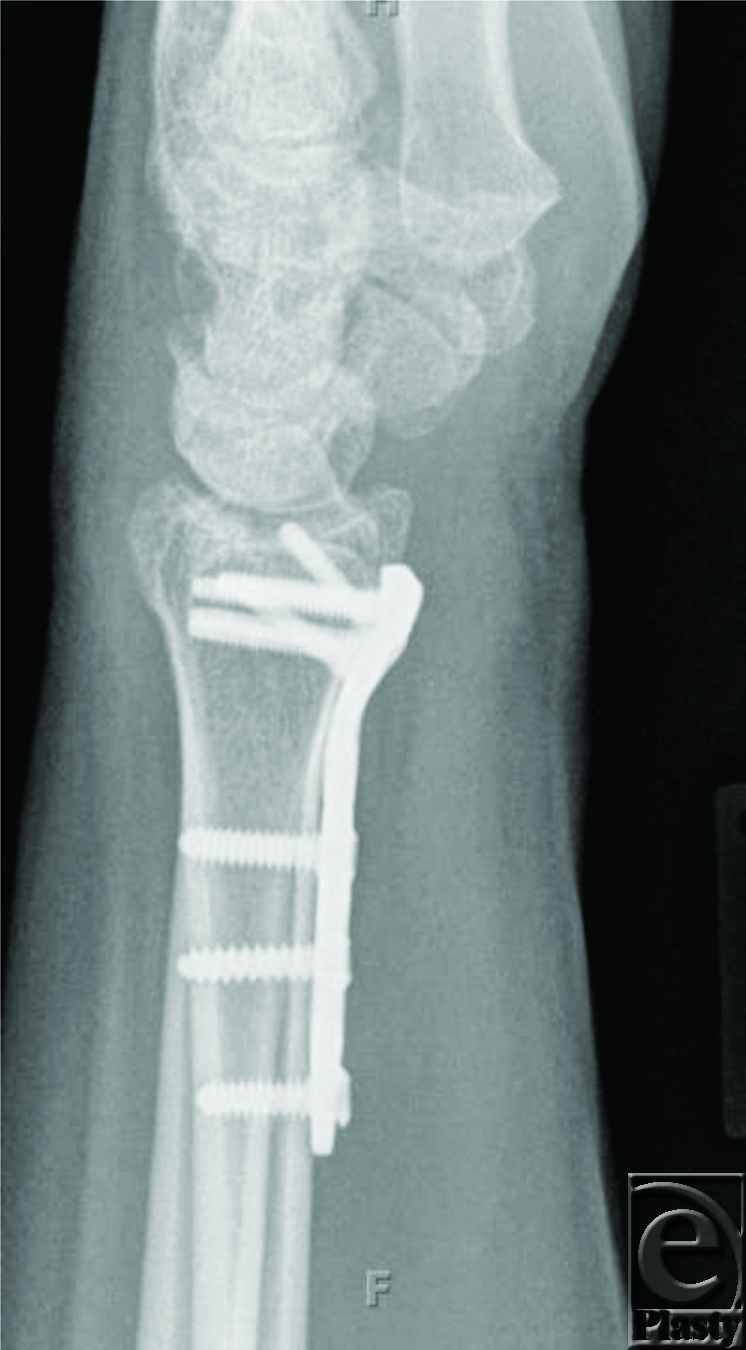
The distal radius plate is in good contact with the bone.

**Figure 3:**
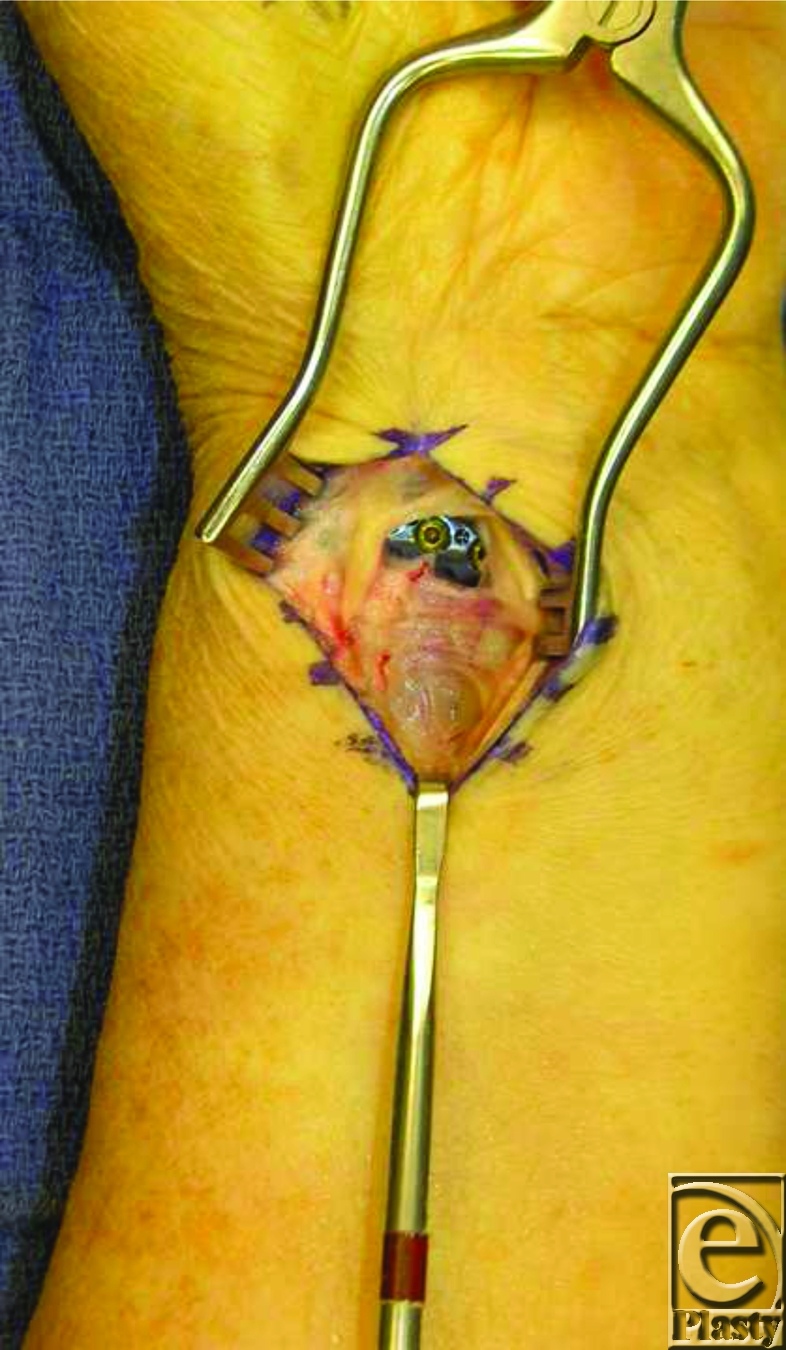
A defect in the pronator quadratus was found in surgery underlying where the FPL tendon passed. More distally and radially, the repair of the pronator to its native bed performed at the end of the first procedure remained intact.

**Figure 4:**
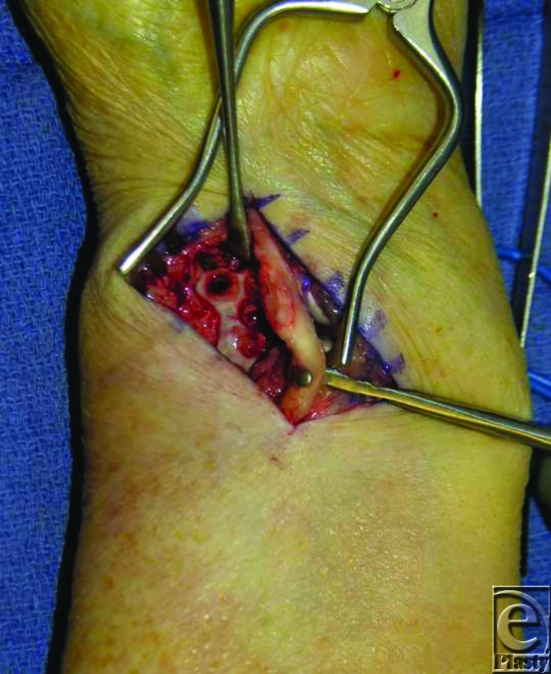
A 20% erosion of the FPL was noted. This was repaired with epitendinous 6-0 polypropylene suture.

Two and half a years later, the patient returned with a 2-month history of volar radial-sided wrist pain. She also complained of a rubbing sensation in this area with thumb motion. On examination, there was palpable crepitus with thumb interphalangeal joint flexion suggesting irritation of the flexor policis longus tendon. The patient was able to actively fire her FPL (Fig 1). The patient was taken for surgical exploration, which revealed mature union of the fracture. The plate was found to be in good contact with bone with no prominent edges (Fig 2). However, an obvious defect in the previously intact pronator quadratus was noted, where it appeared as if the plate had eroded through the muscle. The repair of the connective tissue at the edges of the muscle to the surrounding periosteum/brachioradialis radially and joint capsule distally appeared intact. There was clear demarcation between the intact more proximal muscle fibers and the more distal ruptured area of the muscle (Fig 3). Irritation of the flexor pollicis longus tendon was identified in this location. There was a partial thickness laceration comprising approximately 20% of the tendon substance (Fig 4). This was repaired using 5-0 polypropylene epitendinous sutures. The plate was removed and the remaining intact pronator quadratus was again laid back in its native position.

At final follow-up examination postoperatively, the patient reported complete resolution of her previous volar radial-sided wrist pain. On examination, she had good motion of her thumb interphalangeal joint with no palpable crepitus.

## DISCUSSION

Complication rates as high as 22% and 27% have been reported after volar plating of distal radius fractures.^4^^,^^5^ A recent study of 594 patients reported 13 cases of flexor tendon irritation, with one case of complete tendon rupture occurring 8 months after surgery.^6^ This irritation involved the flexor policis longus and/or flexor digitorum profundus tendons and was observed as late as 41 months after surgery. The authors also identified plate type and surgeon volume as potential risk factors for late complications after volar plate fixation. However, this study made no mention of whether or not the pronator quadratus was laid back over the plate during implantation.

In our practice, the pronator is elevated at the start of the operation to include sufficient connective tissue distally and radially to allow for it to be restored to its native position at the end of the procedure. A small rim of brachioradialis is raised with the muscle radially and a small rim of periosteum is raised with the muscle distally. Maintaining this connective tissue during elevation allows sufficient purchase of sutures to bring the pronator muscle back down to its native position in a manner that provides complete coverage of the underlying hardware at the end of the procedure. Suturing the muscle with the wrist in neutral or even slight pronation also aids with allowing the muscle to be brought back into the appropriate position in a tension-free manner.

In the current patient, we observed flexor pollicis longus irritation despite coverage of the plate with the pronator quadratus at the initial surgery. As with our previously reported patient, she received a second generation, low-profile volar locking plate that was fully in contact with the bone. The current patient had her initial surgery by the senior author, and the pronator was returned to its native position including full coverage of the plate at the end of the procedure. Douthit^7^ observed that the overall bulk of the pronator quadratus is variable, and the muscle can be relatively thin in middle-aged women. This may have been a contributing factor in our case. More research in this area is needed and may lead to further refinements in plate design and surgical technique.

Although we have been unable to definitively prevent flexor tendon irritation after volar plating of distal radius fractures, we do not currently advocate routine removal of this hardware. Despite the 2 cases we have seen, this remains a relatively rare complication.^6^ The risks and operative costs of routinely removing this hardware would outweigh the potential benefits. However, on the basis of our experience with these complications, we stress to all patients at their final postoperative visit that they must inform us of any new increase in pain or crepitus, or change in active range of motion. It is also essential for the surgeon to react quickly to these complaints and potentially intervene before complete tendon rupture occurs.
